# Urolithiasis and sleeve gastrectomy: a prospective assessment of urinary biochemical variables

**DOI:** 10.1590/0100-6991e-20202804

**Published:** 2021-02-18

**Authors:** DENIS WAKED BRITO, FERNANDO SANTA-CRUZ, MARIA AMÉLLIA R AQUINO, WAGNER A NASCIMENTO, ÁLVARO ANTONIO B. FERRAZ, FLÁVIO KREIMER

**Affiliations:** 1 - Hospital das Clínicas da Universidade Federal de Pernambuco (HC/UFPE), Urology Department - Recife - PE - Brasil; 2 - Universidade Federal de Pernambuco (UFPE), Medical course - Recife - PE - Brasil; 3 - Hospital das Clínicas da Universidade Federal de Pernambuco (HC/UFPE), Department of General Surgery - Recife - PE - Brasil

**Keywords:** Bariatric Surgery, Sleeve Gastrectomy, Urine Chemistry, Urinary Lithiasis, Renal Stones, Cirurgia Bariátrica, Gastrectomia Vertical, Química da Urina, Litíase Urinária, Cálculos Renais

## Abstract

**Introduction:**

to evaluate urinary biochemical alterations related to urolithogenesis processes after sleeve gastrectomy (SG).

**Materials and methods:**

*:* prospective study with 32 individuals without previous diagnosis of urolithiasis who underwent SG. A 24-h urine test was collected seven days prior to surgery and at 6-month follow-up. The studied variables were urine volume, urinary pH, oxalate, calcium, citrate, and magnesium and calcium oxalate super saturation (CaOx SS)*.*

**Results:**

patients were mainly women (81.2%), with mean age of 40.6 years. Mean pre- and postoperative BMI were 47.1 ± 8.3 Kg/m^2^ and 35.5 ± 6.1 Kg/m^2^, respectively (p<0.001). Urine volume was significantly lower at the postoperative evaluation in absolute values (2,242.50 ± 798.26 mL x 1,240.94 ± 352.39 mL, p<0.001) and adjusted to body weight (18.58 ± 6.92 mL/kg x 13.92 ± 4.65 mL/kg, p<0.001). CaOx SS increased significantly after SG (0.11 ± 0.10 x 0.24 ± 0.18, p<0.001). Moreover, uric acid levels were significantly lower at the postoperative evaluation (482.34 ± 195.80 mg x 434.75 ± 158.38 mg, p=0.027). Urinary pH, oxalate, calcium, citrate, and magnesium did not present significant variations between the pre- and postoperative period*s.*

**Conclusion:**

SG may lead to important alterations in the urinary profile. However, it occurs in a much milder way than that of RYGB.

## RESUMO

**Introdução:**

avaliar as alterações bioquímicas urinárias relacionadas aos processos de litíase urinária após gastrectomia vertical (GV).

**Método:**

estudo prospectivo, com 32 indivíduos submetidos a GV, sem diagnóstico prévio de urolitíase. Foi coletada urina de 24 horas, sete dias antes da operação e no retorno de 6 meses. As variáveis estudadas foram volume de urina, pH urinário, oxalato, cálcio, citrato e super saturação de oxalato e cálcio (SS CaOx).

**Resultados:**

os pacientes foram em sua maioria mulheres (81,2%), com idade média de 40,6 anos. O IMC médio pré e pós-operatório foi 47,1 ± 8,3 Kg/m^2^ e 35,5 ± 6,1 Kg/m^2^, respectivamente (p<0,001). O volume de urina foi significativamente baixo na avaliação pós-operatória em valores absolutos (2.242,50 ± 798,26 mL versus 1.240,94 ± 352,39 mL, p<0,001) e ajustado ao peso corporal (18,58 ± 6,92 mL/kg versus 13,92 ± 4,65 mL/kg, p<0,001). A SS CaOx aumentou significativamente após a GV (0,11 ± 0,10 versus 0,24 ± 0,18, p<0,001). Além disso, os níveis de ácido úrico apresentaram-se significativamente baixos na avaliação pós-operatória (482,34 ± 195,80 mg versus 434,75 ± 158,38 mg, p=0,027). PH urinário, oxalato, cálcio, citrato e magnésio não apresentaram variações significativas entre os períodos pré e pós-operatório.

**Conclusão:**

a GV pode levar a alterações importantes no perfil urinário. Entretanto, essas ocorrem de forma muito mais leve que na derivação gástrica em Y de Roux.

## INTRODUCTION

The association between obesity and the risk of urinary lithiasis has been extensively reported. The mechanisms underlying this relation are multifactorial^1^ ^,^ ^2^. Studies on the urinary biochemistry of patients with obesity have shown alterations that predispose to the formation of urinary stones, including hypercalciuria, hypocitraturia, hyperoxaluria, hyperuricosuria, and acid pH^3^.

Ironically, patients who undergo surgical treatment for obesity, specifically using procedures with a malabsorptive component, have also an increased risk of urinary lithiasis^4^. This situation occurs mainly due to hyperoxaluria, hypocitraturia, and reduction of urine volume, which leads to an increase in calcium oxalate super saturation (CaOx SS), facilitating the precipitation process^4^ ^-^ ^6^.

Most published articles on the relation between bariatric surgery and urinary lithiasis analyzed patients who underwent Roux-en-Y gastric bypass (RYGB), a procedure with a malabsorptive component responsible for all of the abovementioned urinary biochemical alterations, which increases the risk of urinary stones after surgery^4^ ^,^ ^7^. However, there is a lack of data in the literature regarding urinary lithiasis in patients undergoing non-malabsorptive procedures, especially sleeve gastrectomy (SG). Only a few prospective studies have been published and the results are sometimes conflicting^5^ ^,^ ^7^.

Therefore, this study aims to prospectively evaluate urinary biochemical alterations related to urolithogenesis processes after SG and determine whether this procedure increases or not the risk of formation of urinary stones.

## METHODS

### Study design

We recruited patients who underwent laparoscopic SG in our Institution between July 2018 and December 2019. We included patients from both sexes, aged between 18 and 65, with formal indication for bariatric surgery (BMI between 30 and 34.9kg/m^2^ associated with a severe comorbidity, or BMI between 35 and 40kg/m^2^ associated with any comorbidity, or BMI > 40 kg/m^2^ regardless of comorbidities). Patients with previous diagnosis of urinary lithiasis (regardless of treatment), preoperative glomerular filtration rate < 60mL/min/1.73 m^2^ (calculated using the MDRD equation)^8^, and use of medications that interfered with the urinary metabolism (diuretics, probenecid, angiotensin-converting-enzyme inhibitors, and angiotensin II receptor blockers) were excluded from the study. Patients with inadequate urine collection were also excluded (see “Technical procedures” below).

### Technical procedures

All included patients underwent an abdominal ultrasonography as a screening method for the diagnosis of urinary lithiasis. Those patients who did not present urolithiasis in the ultrasound were evaluated through a 24-h urine collection test at two different moments: seven days prior to surgery and at the 6-month postoperative follow-up. To collect the 24-h urine for testing, patients were instructed to discard the first sample in the morning and collect all subsequent samples, including the first sample in the following morning. Urine samples were stored in a refrigerator (2-8ºC) before sending them to the laboratory for analysis. This occurred, in all cases, right after the collection of the last sample.

To estimate the reliability of the collected samples, 24-h urinary creatinine was measured. The normal 24-h urinary creatinine excretion is 955-2,936mg (or 13-29 mg/kg) for men and 601-1,689mg (or 9-26mg/kg) for women^9^. Levels of urinary creatinine in a 24-h sample below these normal ranges means inadequate urine collection, and thus, the sample was excluded from the analysis. 

The same laboratory analyzed all samples. The quantitative variables were 24-h urine volume, 24-h urine volume adjusted to body weight, urine pH, 24-h levels of urinary oxalate, calcium, citrate, magnesium, uric acid, and calcium oxalate SS, calculated by the Tiselius index^10^.

### Statistical analysis

As part of data analysis, a database was created using Microsoft Excel. It was then exported to SPSS 13.0, in which the analysis was performed. The Kolmogorov-Smirnov test was applied to assess the normality of quantitative variables. To analyze paired samples, the Student T-test was used in situations that had a normal distribution. When the hypothesis of normality of distribution was refuted, the Wilcoxon test was applied. All conclusions considered a significance level of 95%.

## RESULTS

From 51 candidates for SG during the study period, only 41 fulfilled the study criteria. Among these 41 patients, we excluded seven patients from the analysis due to absence of follow-up and two due to severe postoperative complications (gastro-pleural fistula), which needed further interventions. Therefore, only 32 patients completed the pre- and postoperative evaluations with 24-h urine collection tests. We included them in the final analysis. The sample comprised 26 (81.2%) women and six (18.8%) men, with a mean age of 40.6 ± 9.8 years. The participants’ self-declared skin color was predominantly white (75%), followed by brown (22%) and black (3%). The mean preoperative BMI was 47.1 ± 8.3Kg/m^2^ and the mean postoperative BMI was 35.5 ± 6.1 Kg/m^2^ (p<0.001). Approximately 21.8% of the sample presented Type 2 Diabetes Mellitus in the preoperative period and 46.8% presented hypertension.

Table 1 has the values of urinary biochemical variables at pre- and postoperative periods of SG. Urinary creatinine levels certified the reliability of the studied samples. Although some presented a tendency of variation, there was no statistically significant alteration in the values of urinary pH, oxalate, calcium, citrate, and magnesium. Urine volume was significantly lower at the postoperative evaluation in absolute values (2,242.50 ± 798.26 mL x 1,240.94 ± 352.39 mL, p<0.001) and adjusted to body weight (18.58 ± 6.92 mL/kg x 13.92 ± 4.65 mL/kg, p<0.001). CaOx SS increased significantly after SG (0.11 ± 0.10 x 0.24 ± 0.18, p<0.001). Moreover, uric acid levels were significantly lower at the postoperative evaluation (482.34 ± 195.80 mg x 434.75 ± 158.38 mg, p=0.027).

**Table 1:** Biochemical variables of the 24-h urine collection test.

Variable	Pre Mean ± SD	Post Mean ± SD	p-value
Creatinine (mg)	1,302.61 ± 498.37	1,299.53 ± 320.91	0.953*
pH	5.86 ± 0.60	6.02 ± 0.52	0.229**
Volume (mL)	2,242.50 ± 798.26	1,240.94 ± 352.39	< 0.001*
Volume/body weight (mL/kg)	18.58 ± 6.92	13.92 ± 4.65	< 0.001*
CaOx SS#	0.11 ± 0.10	0.24 ± 0.18	< 0.001*
Oxalate (mg)	7.23 ± 4.00	8.32 ± 2.94	0.140*
Calcium (mg)	84.53 ± 62.69	82.03 ± 49.57	0.505*
Citrate (mg)	366.59 ± 282.74	368.66 ± 253.97	0.849*
Magnesium (mg)	51.25 ± 26.35	52.34 ± 20.68	0.708*
Uric acid (mg)	482.34 ± 195.80	434.75 ± 158.38	0.027*

Pre - preoperative evaluation; Post - postoperative evaluation; CaOx SS - calcium oxalate super saturation; (*) Student t test; (**) Wilcoxon test; (#) Tiselius index - AP(CaOx) = 1.9 x Calcium ^*0.84*^ *x Oxalate x Citrate* ^*-0.22*^ *x Magnesium* ^*-0.12*^ *x Volume* ^*-1.03*^ *.*

## DISCUSSION

The onset of urolithiasis in patients undergoing bariatric surgery generally occurs one to two years after the procedure. The mean interval is 1.5-3.6 years between surgery and diagnosis^11^ ^-^ ^14^. Nevertheless, urinary metabolic alterations can be identified earlier, precisely between the 2nd and 6th postoperative months, as Agrawall et al. reported^4^. In our study, 24-h urine samples were collected, and we analyzed them six months after SG. We observed some characteristics that may contribute to the formation of urinary stones, such as an important reduction in urine volume (both absolute values and adjusted to body weight) and an increase in CaOx SS.

Studies analyzing patients undergoing malabsorptive procedures established a correlation between these procedures and, reported the occurrence of lithogenic urinary metabolic alterations^15^ ^,^ ^16^. Espino-Grosso and Canales showed, in a recent review, that patients who undergo RYGB generally present higher levels of urinary oxalate, increased CaOx SS, lower urine volume, and hypocitraturia. These factors cause an increased risk of urolithiasis^7^. Moreover, the authors observed that, apparently, purely restrictive bariatric surgeries do not increase the risk of formation of urinary stones, despite leading to a reduction in 24-h urine volume, which is a potential risk factor for this condition.

Regarding non-malabsorptive bariatric procedures, the literature reports scarce and conflicting results. Some authors have raised the hypothesis that these procedures could also increase the risk of urolithiasis due to the reduction in urine volume^17^. Others have pointed to another direction by suggesting that these procedures would not increase the risk of formation of stones because they do not lead to hyperoxaluria, which is a consequence of malabsorptive operations^18^. Chen et al. found, in a retrospective study with 85 patients followed for 26.8 months after SG, that the incidence of urolithiasis in these patients was very low: only 5.25 per 1,000 person-year^19^. The authors, however, did not study the urinary biochemical profile of their samples. 

In the present study, we observed a significant reduction in total urine volume after SG (2,242.5mL/24-h x 1,240.94mL/24-h, p<0.001). Because the considerable postoperative weight loss, this could have been responsible for these reductions in urine volume, we also analyzed this variable by adjusting it to body weight. We also found a significant decrease after surgery (18.58mL/Kg/24h x 13.92 mL/Kg/24h, p<0.001). These findings are in accordance with previous reports. They can be justified by reduction of water intake, early satiety, and faster gastric emptying, observed after operations with a restrictive component^5^ ^-^ ^7^ ^,^ ^11^ ^,^ ^18^.

CaOx SS is one of the most important variables to assess the risk of CaOx precipitation in urine^20^. The increase in CaOx SS has been largely reported after malabsorptive procedures. It is secondary to increased levels of oxalate and decreased levels of citrate in the urine after the operation^21^. Similarly, in our sample, we observed a significant increase in CaOx SS values (0.11 x 0.24, p < 0.005), however this happened with SG, a non-malabsorptive procedure. Despite this increase, CaOx SS values of all included patients remained below the precipitation threshold (<2) at both pre- and postoperative evaluations. Due to the non-malabsorptive nature of SG, we hypothesized that this increase in CaOx SS may be secondary only to decreased urine volume, which we observed.

Moreover, we found a slight increase in oxalate levels after SG. However, there was no statistical significance (7.23 x 8.32mg/24-h). This was already expected, since hyperoxaluria is a common feature of patients undergoing malabsorptive bariatric surgery^6^ ^,^ ^7^ ^,^ ^22^. The mechanism related to a malabsorptive surgery leading to hyperoxaluria is that the unabsorbed fatty acids bind calcium in the intestines, preventing the formation of gut calcium oxalate, which facilitates the absorption of the unbound oxalate^23^ ^,^ ^24^.

Furthermore, obesity is associated with hypercalciuria. The urinary levels of calcium generally decrease after malabsorptive procedures due to a decreased intestinal absorption of this ion^11^. Durant et al. studied the effects of bariatric surgery on the calcium and thyroid metabolism and found a slight increase in urinary excretion of calcium in RYGB and SG groups, with no statistical significance between them^25^. In addition, there have been no reports of alterations in calcinuria following SG^22^. Our results reinforce this result since there was no statistically significant difference between calcium levels at the pre- and postoperative periods of SG. 

We also analyzed the levels of citrate and magnesium in urine, since both are important urolithogenesis inhibitors acting especially against the formation of calcium crystals^26^ ^,^ ^27^. Despite presenting slightly higher levels at the postoperative period (no statistical significance), both citrate (366.59mg/24-h) and magnesium (51.25mg/24-h) were below ideal levels (640mg/24-h and 70-120mg/24-h, respectively) before and after SG. This could potentially favor the process of formation of stones because there was no reduction in calcium levels in our sample. Our findings are similar as those of Semins et al., who studied a small group of patients undergoing restrictive procedures (gastric banding or SG, and the authors did not find significant alterations in neither citrate nor magnesium in the postoperative period^22^. Conversely, RYGB and other malabsorptive procedures contribute to a significant reduction in citraturia and magnesuria, though presenting decreased levels of calcium in urine, which may balance the processes of inhibition versus formation of crystals^6^ ^,^ ^7^ ^,^ ^11^.

Low urinary pH is a common feature in patients with obesity. Generally, it is attributed to insulin resistance^28^. An acidic urine leads to high concentrations of insoluble uric acid and, consequently, formation of stones^29^ ^,^ ^30^. Moreover, studies reported that patients who underwent bariatric surgery, specifically RYGB, could present a persistent low urinary pH^31^ ^,^ ^32^. On the other hand, our results show that there were no significant alterations in urinary pH after SG, and that its levels remained within the normal range (5.5 - 6.5) during the whole study period. 

Finally, we observed that in both pre- and postoperative periods, urinary uric acid levels remained within the normal range (250-750mg/24-h). However, there was a statistically significant decrease in its levels (482.34 x 434.75mg, p=0.027) in the postoperative period. These findings differ from those of Valezi et al., who observed hyperuricosuria and decreased urinary pH in patients who underwent RYGB^31^. Our results could be explained by significant weight loss alone. However, the real impacts of bariatric surgery are still not completely elucidated, either of RYGB or SG, on uric acid metabolism^32^.

The main limitations of the present study are sample size and the relatively short follow-up period. On one hand, a longer follow-up could provide clinical data regarding the development or not of kidney stones after SG. On the other hand, extending the study period could lead to loss of follow-up, a common problem in prospective studies, which could further reduce our sample. Nevertheless, there are some strengths that deserve attention, especially the well-designed patient selection, excluding individuals who already presented signs of urolithiasis at the preoperative period. The risk of selection bias is thus low.

## CONCLUSIONS

Our results show a significant increase in CaOx SS, despite within the normal range, and an important reduction in urine volume. Yet, the urinary levels of uric acid significantly decreased after SG, which could be a protective factor against formation of uric acid stones in these patients. There are no clear variations in urinary pH, oxalate, calcium, citrate, or magnesium, which can vary following malabsorptive procedures. Therefore, the authors conclude that SG may lead to important alterations in the urinary profile. However, this would occur in a milder way than in RYGB. Randomized controlled trials with longer follow-up periods are further needed to determine the real impacts of SG on formation processes of kidney stones.
